# Immunotherapy in the Treatment of Allergic Rhinitis in Children

**DOI:** 10.7759/cureus.32464

**Published:** 2022-12-13

**Authors:** Ruba A Alamri, Ghaida H Aljabri, Rehab Tahlawi, Hasan A Aljabri

**Affiliations:** 1 Medicine and Surgery, Taibah University, Medina, SAU; 2 Microbiology and Immunology, Faculty of Medicine, Zagazig University, Zagazig, EGY; 3 Allergy and Immunology, King Salman Medical City, Medina, SAU

**Keywords:** allergen-specific immunotherapy (ait), allergic rhinitis (ar), ige, sublingual immunotherapy (slit), subcutaneous immunotherapy (scit), children

## Abstract

Allergic rhinitis (AR) is an inflammation of the nasal membranes characterized by multiple allergic symptoms. It is a widespread health problem that affects patients’ ability to engage in social and physical activity, which lowers their quality of life. The pathophysiology of AR is complex and requires sensitization and the development of a specific immune response to the allergen. Allergen‐specific immunotherapy (AIT) is a therapeutic method that induces specific immune tolerance to allergens.

The objectives of this review are to demonstrate the mechanism of action of immunotherapy, explain how it alleviates clinical symptoms of allergic rhinitis, list the indications and contraindications of immunotherapy in the treatment of allergic rhinitis, and identify different modalities of allergen immunotherapy, their disease-modifying effects, as well as their potential risks and benefits.

The review of the literature highlights that T-cell and B-cell responses to inhaled allergens are altered by AIT, which decreases both early and late reactions to allergen exposure. To induce clinical and immunologic tolerance, especially in the pediatric age, escalating dosages of the causing allergen are administered subcutaneously or sublingually. AIT is indicated for severe persistent AR when avoidance measures and medications are inadequate to control the symptoms.

To conclude, AIT is a disease-modifying therapy that is safe and effective for the treatment of allergic rhinitis. It is indicated when the symptoms are uncontrolled or when there are undesirable effects from pharmacotherapy.

## Introduction and background

One of the most common allergic diseases around the world is allergic rhinitis (AR). For the last few decades, studies have shown that its prevalence has increased all over the world. Among Saudi children, it has been reported to be as high as 26.51% [1].

Untreated AR can have enormous negative consequences, particularly in children, since it is associated with numerous complications and comorbidities such as asthma, otitis media, sinusitis, nasal polyps, as well as lower respiratory tract infections [2-3].

Clinical symptoms, such as sneezing, itching, rhinorrhea, and nasal congestion, with no signs of lower respiratory tract infections or anatomic abnormalities of the nose, are diagnostic indicators of AR [3]. This is associated with a positive prick test and a high level of specific immunoglobulin E (IgE) to confirm the correlation between clinical history and potential allergens. The patient’s history, as well as the family history, are important in diagnosis, as there is a strong genetic component of the allergic response [4-5].

Treatment of AR depends on the severity of the symptoms and their impact on daily activities. The initial treatment includes patient education and allergen avoidance, in particular, house dust mites, fungi, pets’ hair, dander, grass, trees, indoor plants, and grass pollens, as well as other allergy-causing substances [2,4-6].

Oral antihistamines, topical decongestants, and inhaled corticosteroids are pharmacological options for the treatment of AR. In some cases, oral corticosteroids may also be prescribed. In spite of the efficacy of pharmacological approaches in improving the outcome of allergic rhinitis, they fail to modify the underlying immune response [2,4,7].

Allergen-specific immunotherapy (AIT) is the only disease-modifying therapy able to change the natural history of IgE-mediated allergic diseases [8]. Using this method, a specific allergen is slowly and incrementally administered; as a result, clinical symptoms are alleviated, the severity of the disease is reduced, and its progression is prevented [2,7-9].

AIT is a therapeutic method recommended for patients with moderate or severe persistent AR that is not responding to usual treatment. It can be administered through different routes, including primarily sublingual (sublingual immunotherapy (SLIT)), and subcutaneous (subcutaneous immunotherapy (SCIT)) routes. Significant progress has been made in terms of AIT efficacy and safety [10].

Our aim is to list the indications, demonstrate the mechanisms of action of immunotherapy in the treatment of allergic rhinitis, as well as identify different modalities of allergen immunotherapy, including their disease-modifying effects, potential risks, and benefits among children. As regards methodology, we searched for articles on PubMed and Google Scholar. Research that was outdated was excluded. Articles that met the inclusion criteria were included in this research.

## Review

Pathophysiology of allergic rhinitis

Early- and late-phase allergic reactions are both parts of the complex pathophysiology of AR. In pre-sensitized individuals, exposure to allergens such as pollens, mites, or animal dander is identified by antigen-specific IgE fixed on receptors on mast cells and basophils and triggered the process [11-12].

Mast cell degranulation is a defining feature of the early-phase response [13]. The fast onset of acute nasal symptoms (such as sneezing and rhinorrhoea) over a matter of minutes and the appearance of ocular symptoms (i.e., tearing, redness, and itching) are features of this phase. Histamine release mainly from nasal cell mucosa is what causes these symptoms. The early-phase histamine release along with the actions of other strong proinflammatory cytokines (like leukotrienes) and eicosanoids (including prostaglandins and kinins) lead to an increase in vascular permeability, which results in the production of edema [13].

After exposure to an allergen, the late-phase reaction takes many hours to manifest. Cellular recruitment of basophils, neutrophils, T-lymphocytes, monocytes, and eosinophils, as well as the release of numerous mediators, such as cytokines, prostaglandins, and leukotrienes, which support the inflammatory response, are characteristic of this phase [11-12].

Nasal congestion develops and persists as a result of this late-phase inflammatory reaction, along with further tissue edema and tissue remodeling [14-15]. Tissues become primed and react more forcefully to allergen exposure as a result of mucosal inflammation. Furthermore, bronchial hyper-responsiveness occurs as a result of these late-phase responses and changes in tissue responsiveness [13].

Allergen-specific immunotherapy

Allergen-specific immunotherapy refers to giving a patient increasing doses of an allergen to which they have type I acute hypersensitivity. When exposed to that allergen, the patient is protected from the inflammatory reactions and allergy symptoms that are related to that exposure [16].

Mechanism of action of immunotherapy

Since it was developed more than 80 years ago, AIT is frequently used to treat allergic illnesses. Numerous immunological alterations have been seen both during and after immunotherapy clinical courses, and a variety of mechanisms have been proposed to explain these beneficial clinical benefits. But it is still unknown which, if any, of these immunological alterations actually contribute to the mechanisms by which this therapeutic approach reduces the patient's symptoms [17].

1) Reduction of Specific Immunoglobulin E (IgE)

The development of airway allergic diseases like AR and asthma requires interaction between an environmental allergen and its particular IgE antibody. Increased development of specific IgE antibodies against one or more environmental allergens, such as pollen, house dust mites, and fungal spores, following the first exposure to such allergens, is the first immunopathogenesis necessary. IgE molecules bind to receptors on the surface of circulating basophils and tissue mast cells with specificity and avidity. When an individual is exposed to the same allergen(s) a second time, the antigen interacts with a limited number of surface-bound IgE molecules, causing these molecules to cross-link. This activation causes basophils and mast cells to generate and release a number of powerful mediators that cause clinical allergies. The clinical course might improve if the allergen-specific IgE antibodies are reduced. Therefore, it can be suggested that immunotherapy acts by having a negative impact on the synthesis of IgE specific to allergens [17].

2) Immune Tolerance

Subcutaneous (SCIT) or sublingual (SLIT) allergy-specific immunotherapy (AIT) is becoming increasingly common [18]. To employ AIT in children effectively, guidelines have been created [19].

The SCIT/SLIT immunological tolerance process in allergic rhinitis is intricate and poorly understood at this time. It has been proposed that AIT repairs the immunological disparity by converting the cytokine pattern from the Th2 (IL-4, IL-5, IL-13) to the Th1 (IL-2, IFN-γ) type. In addition, AIT reduces the production of allergen-specific IgE and at the same time induces the production of IgG4-blocking antibodies. AIT was found also to activate the immunosuppressive Treg (T regulatory) cells, resulting in allergen tolerance [20].

Unfortunately, there is a lack of accurate biomarkers that may be used to select patients for AIT and track the effectiveness of the treatment. An essential cytokine of the T helper 2 (Th2) immune response that predominates in allergic diseases, such as allergic rhinitis, is interleukin-33 (IL-33) [21]. Previous studies have demonstrated that blocking the interaction of IL-33/ST2 alleviates the severity of allergic disease by reducing Th2 cytokine production, eosinophilic inflammation, serum IgE level, and airway hyper­reactivity [22-25]. The level of IL-33 has been correlated with the severity of allergic rhinitis [26]. As a result, scientists suggest that IL-33 may serve a specific role in monitoring AIT, especially SLIT [27]. Also, IL-13 is being investigated as a potential biomarker for the reaction to and monitoring of AIT in allergic rhinitis [20].

AIT is the sole disease-modifying therapy among all the potential treatments for IgE-mediated allergy disorders that have been proposed. Despite the poorly understood mechanism of action, this effect may be related to the restriction of inflammatory cells' migration to tissues, early desensitization of mast cells and basophils, reduced basophil activity in the circulation, and decreased numbers and activities of effector cells in the mucosal lining of target organs such as mast cells, basophils, and eosinophils [10].

Indications and contraindications of AIT

Candidates for AIT include patients with clinically obvious IgE-mediated AR, allergic asthma, allergic conjunctivitis, and Hymenoptera sensitivity. In order to use immunotherapy, it is necessary to identify specific IgE for the target allergens and show a correlation between symptoms and exposure to those allergens [16,27].

When symptoms of allergic respiratory disorders in children continue despite efforts to avoid allergens, AIT is also recommended as a therapeutic option. Children who have a positive skin prick test or IgE against one or more allergens and whose exposure is linked to moderately severe AR symptoms, with or without conjunctivitis interfering with daily activities or sleep, and/or allergic asthma, are eligible for AIT [28].

Poor symptom control despite medication or in situations where avoidance is impossible is another rationale for AIT. AIT should also be taken into account when medication manages symptoms but patients want to avoid long-term treatment or in patients who frequently have major adverse drug reactions. Additionally, the patient's readiness needs to be taken into account [19].

The relative contraindications to allergen immunotherapy include any medical condition that reduces the patient’s capacity to survive a systemic allergic reaction such as severe or uncontrolled asthma and significant cardiovascular disease. Furthermore, AIT is not recommended for chronic urticaria and/or angioedema. Regarding food allergy, using AIT is still under investigation (Table 1) [27]. 

**Table 1:** Indication and contraindication of AIT in children The information used in this table was obtained from Carlo Caffarelli et al. [8]. AIT: allergen-specific Immunotherapy

Indication of AIT	Contraindication of AIT
Persistent symptoms	Uncontrolled asthma
Moderate to severe allergic rhinitis with or without conjunctivitis and positive skin prick test or IgE for one or more allergens	Cardiovascular diseases
Adverse events elicited by drugs	precaution to patients receiving b-blockers or ACEi angiotensin-converting enzyme inhibitors

Allergen-specific immunotherapy modalities

The availability, cost, tolerability (better for SLIT), patient choice (injections are less tolerated in small children), and adherence are all aspects that should be taken into account when determining the best method of AIT administration (higher for SCIT beyond pediatric age). It's crucial to note, nevertheless, that meta-analyses on AIT do not believe that safety and efficacy depend on the product selected for therapy. To prevent decision-influencing generalizations about administration methods or age groups, each product should be examined independently [10].

Based on cutaneous testing of the patient with several allergens, the allergen delivered in AIT should be suitably selected. Therefore, it's crucial to be knowledgeable about the main aeroallergens in a patient's area [29].

Typically, antigen mixes are injected into the subcutaneous tissues SC. This kind of delivery is referred to as SCIT, which was the initial method of administering AIT. For allergens like grass, house dust mites, and tree pollen, it is available [30].

The building phase of SCIT began with SC injections of the inhaled allergens once or twice a week, either as aqueous allergen vaccines, depot formulations of chemically altered allergens (allergoids), or physically adsorbed allergens (depots). It is administered in extremely small dosages that are unlikely to result in anaphylaxis. Over several months, these doses are gradually raised until they are at an effective level. At this moment, the maintenance phase begins and lasts typically for three to five years [29,31].

SLIT, in addition to SCIT, is currently the widely used strategy [32]. In seasonal AR, SLIT has been demonstrated to lessen symptoms by 30-40% and prescription use [16]. SLIT varies depending on the formulation, but pills are typically taken once daily beginning three to four months before the pollen season [2].

SLIT involves swallowing an aqueous solution that has been placed beneath the tongue for two minutes to expose the patient to the allergen [33]. The patient should receive the initial dose of SLIT while being monitored by a doctor for 30 minutes. Thereafter, the patient can receive successive doses at home [34]. Although research on the ideal treatment period is ongoing, at least three years of therapy are advised [35]. Unquestionably, further years of SLIT may help maintain the treatment result, which, in grass pollen trials, persisted for at least two years after therapy was stopped [2].

SLIT has an advantage over SCIT because of its improved safety and ease of administration in pediatrics. Additionally, it helps prevent the discomfort of injections and the visits that are necessary for allergy shots [33]. Recently, SLIT tablets for treating house dust mites (HDM) and grass pollen (GP) received certification for use in children, adolescents, and adults [10].

There is clear evidence that SLIT is effective in dramatically lowering nasal and ocular symptoms, medication use, and symptom­medication score in children with allergic rhinoconjunctivitis [28,35-38]. Children's use of SLIT for pre/costagional and continuous schedules of perennial and seasonal allergies has been demonstrated [37]. SLIT prevents AR from developing into asthma and may lessen recurrent sensitizations [37,39-40].

It has been observed that SLIT and SCIT are useful for treating asthma and rhinoconjunctivitis brought on by inhalant allergens. According to meta-analyses comparing SCIT and SLIT, SCIT is more effective while SLIT is safer (Table 2) [16,35].

**Table 2:** Main features that clinically distinguish SCIT from SLIT The information used in this table was obtained from Carlo Caffarelli et al. [8]. SCIT: subcutaneous immunotherapy. SLIT: sublingual immunotherapy

SCIT	SLIT
Higher adherence for SCIT beyond pediatric age	Improved safety
Greater experience in its use ( historically the first and most used route of administration)	The patient burden is lowered because injections are not required. helps to minimize the discomfort of office visits
Some evidence of greater efficacy is yet to be confirmed	Avoid the inconvenience of office visits

Potential benefits of allergen immunotherapy

As children get older, the allergic march may advance, making allergic illnesses a unique concern in the pediatric age group [41-43].

Other problems are related to long-term pharmacotherapy. Certain factors such as the age of the child and a lack of understanding about the disease, treatment, or schedule of medications make the children fully dependent on their caregivers to administer their medications. Hence, the problem of non-compliance appears beside the difficulties in correctly delivering inhaled drugs [44].

As a result, the quality of life of the children themselves and their parents is usually affected by increasing the number of emergency department visits and hospitalization and healthcare costs. On the academic side, this leads to frequent absenteeism and impaired school performance [45-46].

Many clinical trials and meta-analyses have convincingly demonstrated the effectiveness of AIT in reducing symptoms of AR and drug consumption, with a consequent improvement of the overall quality of life and avoidance of the above-mentioned drawbacks of long-term pharmacotherapy [47].

Potential side effects of allergen immunotherapy

Although AIT has been shown to be safe in both adults and children, it could produce some side effects. Most of these side effects are local and less serious [31]. Oropharyngeal signs and symptoms and gastrointestinal reactions are some of the local effects of SLIT that have a wide range of prevalence, going from 50% to 85% [48]. Other less serious medical concerns related to SCIT include needle phobia, local pain from the injection, and problems with lymphatic drainage in post-mastectomy patients [31].

## Conclusions

AIT is a therapeutic method for atopic diseases, including allergic rhinitis, in which the allergen is introduced incrementally and gradually, resulting in a relief of clinical symptoms, a decrease in the severity of the disease, and the prevention of disease progression, particularly in the pediatric age group, where allergic diseases are a particular issue. The patient may administer AIT sublingually at home or subcutaneously in the doctor's office according to many factors, including availability, cost, tolerability, patient preference, and adherence.

AIT is indicated to reduce the morbidity from AR when the symptoms of allergy are uncontrolled despite pharmacotherapy and avoidance measures or in the case of the experience of undesirable adverse effects from pharmacotherapy. AIT decreases the sensitivity to allergens and often leads to lasting relief of allergy symptoms even after treatment is stopped, and it can prevent the progression of allergic disease from AR to asthma in children. This makes it a cost-effective, beneficial treatment approach for many people.
